# Supplementary material for: Chemosensitivity of 3D Pancreatic Cancer Organoids Is Not Affected by Transformation to 2D Culture or Switch to Physiological Culture Medium
Source: Cancers (Basel). 2022 Nov 16;14(22):5617. doi: 10.3390/cancers14225617 (PMC9688175; doi:10.3390/cancers14225617)
Supplement: Supplementary file 1 [file cancers-14-05617-s001.zip › cancers-2000392-supplementary.pdf]

cancers

MDPI

Supplementary Materials: Chemosensitivity of 3D Pancreatic Cancer Organoids Is not Affected by Transformation to 2D Culture or Switch to Physiological Culture Medium

Vincent Gassl, Merel R. Aberle, Bas Boonen, Rianne D. W. Vaes, Steven W. M. Olde Damink and Sander S. Rensen

Table S1. Comparison of human plasma composition with Plasmax and DMEM/F-12.

Proteinogenic Amino Acids	Human Plasma	Plasmax™	DMEM/ F-12
L-Alanine	230 - 510	510	50
L-Arginine	13 - 64	64	699
L-Asparagine	45 -130	41	50
L-Aspartic acid	0 - 6	6	50
L-Cysteine	23.2 - 43.8	33	100
L-Glutamate	32 -140	98	50
L-Glutamine	420 -720	650	2500
Glycine	170 - 330	330	250
L-Histidine	26 - 120	120	150
L-Isoleucine	42 - 100	140	416
L-Leucine	66 - 170	170	451
L-Lysine	150 - 220	220	499
L-Methionine	16 - 30	30	116
L-Phenylalanine	41 - 68	68	215
L-Proline	110 -360	360	150
L-Serine	56 - 140	140	250
L-Threonine	92 - 240	240	449
L-Tryptophan	44.8 - 64.2	78	44
L-Tyrosine	45 - 74	74	214
L-Valine	150 - 310	230	452
**Non-proteinogenic Amino Acids**			
α-Aminobutyrate	15 - 41	41	
L-Citrulline	16 - 55	55	
L-Cystine	30 - 65	65	100
L-Homocysteine	6.1 - 12.1	9	
4-Hydroxy-L-proline	3 - 23	13	
L-Ornitine	27 - 80	80	
L-Pyroglutamate	12.2 - 15.3	20	
**Amino Acids Derivatives**			
L-Acetyl glycine	69.7	70	
L-Carnosine	5.5 - 7.5	6	
Glutathione (reduced)	32.2 - 41.8	37	
Putrescine	0.1 - 0.3		0.5
Taurine	45 - 130	130	
N-Trimethylglycine (betaine)	49.6 - 94.4	72	

**Other Components**			
Acetate	26.8 - 57	42	
Acetone	24.8 - 84	55	
Acetyl carnitine	2.5 - 8.6	5	
Citrate	87.2 - 141.2	114	
Carnitine	34.1 - 57.3	46	
Creatine	8.4 - 65	37	
Creatinine	60.5 - 87.7	74	
Formate	19.5 - 46.1	33	
Fructose	28.0 - 34.0		
Galactose	53.6 - 123		
D-Glucose	4598.5 - 5344.1	5560	17506
	**Human Plasma**	**Plasmax™**	**DMEM/ F-12**
Glycerol	331.2 - 532	82	
2-Hydroxybutyrate	23.5 - 39.1	31	
3-Hydroxybutyrate	10.6 - 143.2	77	
3-Hydroxyisobutyrate	19.0 - 23.0	20	
Hypoxanthine	4.5 - 5.3	5	15
Lactate	1118.2 - 1860.6	500	
Linoleic Acid	45.8 - 121.8		0.15
Lipoic Acid	0.060 - 0.094		0.5
Malonate	12.3 - 14.7		
Methyl acetoacetate	for acetoacetate 4.1 - 77.1	41	
Phenol Red		25.0	21.5
Pyruvate	9.3 - 59.7	100	500
Succinate	23.5	23	
Thymidine	0.1 - 0.3		1.5
Uracil	1.1 - 3.1	2	
Urate	228.9 - 315.1	270	
Urea	3920.4 - 8228.8	3000	
Uridine	1.8 - 4.4	3	
**Inorganic Salts**			
Ammonium Chloride	for NH_4^+ for Cl^-	50	
Calcium Chloride	for Ca^{2+} and Cl^-	1800	1050
Calcium Nitrate	for Ca^{2+} for NO_3^-		
Magnesium Chloride	for Mg^{2+} and Cl^-		302
Magnesium Sulfate	for Mg^{2+} and SO_4^{2-}	813	407
Potassium Chloride	for K^+ and Cl^-	5330	4157
Potassium Nitrate	for K^+ for NO_3^-		
Sodium Bicarbonate	for Na^+ and HCO_3^-	26191	29024
Sodium Chloride	for Na^+ and Cl^-	118706	120612
Sodium Phosphate monobasic	for Na^+ and PO_4^{3-}	1010	453
Sodium Phosphate dibasic	for Na^+ and PO_4^{3-}		500
**Trace Elements**			
Ammonium Metavanadate	for NH_4^+ for V	0.0026	
Cupric Sulfate	for Cu for SO_4^{2-}	0.0052	0.0052
Ferric Chloride	for Fe for Cl^-		
Ferric Nitrate	for Fe for NO_3^-	0.1238	0.1238

Ferric Sulfate	for Fe for SO_4^{2-}	1.0428	1.5000
Manganous Chloride	for Mn for Cl^-	0.0002	
Sodium Selenite	for Na^+ for Se	0.0289	
Zinc Sulfate	for Zn for SO_4^{2-}	1.50	1.50
**Vitamins**			
p-Aminobenzoate	5.0 - 32.0		
Ascorbate	57.9 - 67.3	62	
D-Biotin	0.0006 - 0.0019	4.100	0.014
Choline	9.2 - 19.8	7.1	64.1
Folate	0.017 - 0.025	2.30	6.00
myo-Inositol	17.1	11.1	70.0
Niacinamide	0.435 - 0.445	8.2	16.6
D-Calcium pantothenate	4.5 - 5.3	2.10	4.70
Pyridoxine	0.007 - 0.060	4.90	9.80
Riboflavin	0.0054 - 0.028	0.30	0.58
Thiamine	0.078 - 0.114	3.0	6.4
Vitamin B12	0.00017 - 0.00033	0.0050	0.5000

Adjusted from Tobias Ackermann and Saverio Tardito [16].

Table S2. Media composition of Tumor 2 medium.

Growth factor	Final conc. T2
Adv. DMEM/F12+++ /	base
Wnt3a	50 % (v/v)
Noggin	10% (v/v)
Rspondin1	10% (v/v)
B27 supplement (B27)	1:50
N-acetyl-cysteine (nAc)	1.25 mM
Primocin	1:500
Nicotinamide (N)	10 mM
A83.01 (T)	500 nM
FGF10 (F)	100 ng/mL
Gastrin (G)	10 nM
Rho kinase Inhibitor Y-27632 (Y)	10 μM

Table S3. Medium composition of Tumor 2 and Plasmax Tumor 2 media for 2D organoids .

Growth factor	Final conc. 2DT2/2DPlxT2 (adv.DMEM/F-12 / Plasmax)
Adv. DMEM/F12+++ /Plasmax™+++	base
Wnt3a	25 % (v/v)
Noggin	5% (v/v)
Rspondin1	5% (v/v)
B27 supplement (B27)	1:100
N-acetyl-cysteine (nAc)	0.625 mM
Primocin	1:1000
Nicotinamide (N)	5 mM
A83.01 (T)	250 nM
FGF10 (F)	50 ng/mL
Gastrin (G)	5 nM

Rho kinase Inhibitor Y-27632 (Y)	5 μM

Table S4. Proliferation metrics of PANCO09b and PANCO11b cultured in 3D versus 2D.

Cell culture	Parameter	3D	2D	p-value
**PANCO09b**	doubling time (h)	75.85 ± 22.76	74.73 ± 1.810	0.9364
	growth rate/ day	0.236 ± 0.085	0.222 ± 0.005	0.7863
**PANCO11b**	doubling time (h)	80.26 ± 40.36	52.35 ± 6.09	0.3051
	growth rate/ day	0.237 ± 0.119	0.320 ± 0.035	0.2984

Data are presented as mean ± SD of three independent experiments performed in triplicate. Independent sample t-test was used to assess differences. Significance was reached when $p < 0.05$.

Table S5. Differentially expressed genes between 3D organoids and corresponding 2D transformed cell cultures.

Name	Ensembl ID	Function [19–21]
**None**	ENSG00000263244	• RNA gene
**RSC1A1**	ENSG00000215695	• involved in Glucose cotransporter 1 inhibition
**HERC2P4**	ENSG00000230267	• Pseudogene
**LY75-CD302**	ENSG00000248672	• Unknown function
**None**	ENSG00000260772	• RNA gene
**ATP2A3**	ENSG00000074370	• Magnesium-dependent ATPase<br>• Calcium transporter
**None**	ENSG00000273489	• RNA gene
**CDKL3**	ENSG00000006837	• Cyclin dependent protein kinase<br>• Regulator of cell cycle progression
**PSAPL1**	ENSG00000178597	• Lysosomal degradation of sphingolipids
**GCA**	ENSG00000115271	• Calcium-binding protein<br>• Abundant in neutrophils and macrophages
**AQP12B**	ENSG00000185176	• Water and small neutrals carrier
**SLC13A2**	ENSG00000007216	• Cotransport of sodium ions and dicarboxylates such as succinate and citrate
**MIR17HG**	ENSG00000215417	• RNA gene
**RBP4**	ENSG00000138207	• Retinol-binding protein<br>• Transports retinol from liver storage through plasma
**IHH**	ENSG00000163501	• Intercellular signaling<br>• Involved in events during development
**R3HDML-AS1**	ENSG00000226812	• RNA gene
**JAML**	ENSG00000160593	• transmembrane protein of leukocytes<br>• leukocyte migration
**ADGRV1**	ENSG00000164199	• G-protein coupled receptor<br>• involved in development of hearing and vision
**HOXC10**	ENSG00000180818	• Sequence-specific transcription factor<br>• Developmental regulatory system
**MUC5AC**	ENSG00000215182	• Gel forming glycoprotein<br>• Gastric and respiratory tract<br>• Protects mucosa from infection
**LINC01301**	ENSG00000251396	• RNA gene
**GPI**	ENSG00000105220	• Glycolytic enzyme<br>• Catalyzes G-6-P → F-6-P
**PLA2G4F**	ENSG00000168907	• Lipase<br>• Potential role in membrane lipid remodeling and biosynthesis of lipid mediators

Name	EnsemblID	• Function [19–21]
**OTC**	ENSG00000036473	• Enzyme involved in urea cycle
**None**	ENSG00000224739	• RNA gene
**GORASP2**	ENSG00000115806	• Involved in membrane stacking of Golgi cisternae<br>• Involved in process by which Golgi stacks reform after breakdown during mitosis and meiosis<br>• Required for acrosome formation during spermiogenesis
**MON1B**	ENSG00000103111	• Involved in early viral transcription
**NDRG1**	ENSG00000104419	• Stress responsive protein involved in hormone response, cell growth, and differentiation<br>• Tumor suppressor<br>• Necessary but not sufficient for p53/TP53-mediated caspase activation and apoptosis<br>• Role in cell trafficking
**IL1R2**	ENSG00000115590	• Interleukin receptor
**MYCN**	ENSG00000134323	• Member of MYC family<br>• Located in nucleus<br>• Amplification associated with variety of tumors
**FFAR4**	ENSG00000186188	• G-protein coupled receptor involved in adipogenesis, energy metabolism, inflammation

Table S6. IC_{50} values of PANCO09b and PANCO11b cultured in 2D versus 3D exposed to gemcitabine, paclitaxel, SN-38, 5-FU, and oxaliplatin.

Cell culture	Drugs	IC_{50} (μM) ± SD Adv. DMEM/F-12 2D	3D	*p*-value
**PANCO09b**	**Gemcitabine**	$6.24 * 10^{-3} \pm 0.50 * 10^{-3}$	$8.12 * 10^{-3} \pm 4.24 * 10^{-3}$	0.323
	Paclitaxel	$1.66 * 10^{-3} \pm 0.51 * 10^{-3}$	$1.07 * 10^{-3} \pm 0.79 * 10^{-3}$	0.342
	SN-38	$5.3 * 10^{-3} \pm 0.76 * 10^{-3}$	$4.25 * 10^{-3} \pm 0.88 * 10^{-3}$	0.128
	5-FU	13.20 ± 1.44	11.87 ± 7.84	0.836
	Oxaliplatin	10.38 ± 0.69	8.61 ± 0.91	0.170
**PANCO11b**	Gemcitabine	$6.02 * 10^{-3} \pm 2.15 * 10^{-3}$	$6.87 * 10^{-3} \pm 1.24 * 10^{-3}$	0.656
	Paclitaxel	$2.20 * 10^{-3} \pm 0.67 * 10^{-3}$	$4.48 * 10^{-3} \pm 2.46 * 10^{-3}$	0.200
	SN-38	$8.20 * 10^{-3} \pm 2.31 * 10^{-3}$	$1.13 * 10^{-3} \pm 0.59 * 10^{-3}$	0.027
	5-FU	14.21 ± 3.41	13.25 ± 6.31	0.867
	Oxaliplatin	5.36 ± 0.86	32.30 ± 32.34	0.212

Data are presented as mean ± SD of three independent experiments performed in triplicate. Independent sample t-test was used to assess differences. Significance was reached when $p < 0.05$.

Table S7. GR_{50} values of PANCO09b and PANCO11b 2D versus 3D exposed to gemcitabine, paclitaxel, SN-38, 5-FU, and oxaliplatin.

Cell culture	Drugs	GR50 (μM) ± SD Adv. DMEM/F-12 2D	3D	*p*-value
PANCO09b	Gemcitabine	$6.24 * 10^{-3} \pm 0.50 * 10^{-3}$	$7.44 * 10^{-3} \pm 6.09 * 10^{-3}$	0.595
	Paclitaxel	$1.66 * 10^{-3} \pm 0.51 * 10^{-3}$	$0.87 * 10^{-3} \pm 0.60 * 10^{-3}$	0.143
	SN-38	$5.3 * 10^{-3} \pm 0.76 * 10^{-3}$	$1.96 * 10^{-3} \pm 1.43 * 10^{-3}$	0.076
	5-FU	13.20 ± 1.44	5.24 ± 3.61	0.061
	Oxaliplatin	11.67 ± 2.47	6.53 ± 3.10	0.128
PANCO11b	Gemcitabine	$7.42 * 10^{-3} \pm 1.75 * 10^{-3}$	$2.55 * 10^{-3} \pm 2.20 * 10^{-3}$	0.069
	Paclitaxel	$3.48 * 10^{-3} \pm 0.99 * 10^{-3}$	$1.41 * 10^{-3} \pm 1.23 * 10^{-3}$	0.126
	SN-38	$9.82 * 10^{-3} \pm 0.71 * 10^{-3}$	$3.80 * 10^{-3} \pm 0.26 * 10^{-3}$	0.001
	5-FU	44.85 ± 12.23	11.90 ± 0.99	0.063
	Oxaliplatin	28.65 ± 15.49	6.11 ± 3.08	0.181

Data are presented as mean ± SD of three independent experiments performed in triplicate. Independent sample t-test was used to measure differences. Significance was reached when $p < 0.05$.

Table S8. Doubling time and growth rate of PANCO09b and PANCO11b growing in conventional versus physiological medium.

Cell culture	Parameter	Adv.DMEM/F-12	Plasmax	*p*-value
PANCO09b	doubling time (h)	74.73 ± 1.81	63.36 ± 3.67	< 0.001
	growth rate/ day	0.222 ± 0.005	0.263 ± 0.014	< 0.001
PANCO11b	doubling time (h)	52.35 ± 6.09	62.68 ± 2.89	0.057
	growth rate/ day	0.320 ± 0.035	0.266 ± 0.012	0.063

Data are presented as mean ± SD of three independent experiments performed in triplicates. Independent sample t-test was used to measure differences. Significance was reached when $p < 0.05$.

Table S9. IC_{50} values of PANCO09b and PANCO11b growing in conventional versus physiological media exposed to gemcitabine, paclitaxel, SN-38, 5-FU, and oxaliplatin.

Cell culture	Drugs	IC50 (μM) ± SD Adv.DMEM/F-12	Plasmax	*p*-value
PANCO09b	Gemcitabine	$6.24 * 10^{-3} \pm 0.50 * 10^{-3}$	$8.12 * 10^{-3} \pm 4.24 * 10^{-3}$	0.488
	Paclitaxel	$1.66 * 10^{-3} \pm 0.51 * 10^{-3}$	$1.45 * 10^{-3} \pm 0.51 * 10^{-3}$	0.647
	SN-38	$5.3 * 10^{-3} \pm 0.76 * 10^{-3}$	$4.25 * 10^{-3} \pm 0.88 * 10^{-3}$	0.177
	5-FU	13.20 ± 1.44	11.55 ± 1.32	0.355
	Oxaliplatin	10.38 ± 0.69	7.68 ± 3.39	0.247
PANCO11b	Gemcitabine	$6.02 * 10^{-3} \pm 2.15 * 10^{-3}$	$5.28 * 10^{-3} \pm 0.69 * 10^{-3}$	0.602
	Paclitaxel	$2.20 * 10^{-3} \pm 0.67 * 10^{-3}$	$1.55 * 10^{-3} \pm 0.35 * 10^{-3}$	0.211
	SN-38	$8.20 * 10^{-3} \pm 0.23 * 10^{-3}$	$5.46 * 10^{-3} \pm 0.78 * 10^{-3}$	0.123
	5-FU	14.21 ± 3.41	11.42 ± 6.82	0.656
	Oxaliplatin	5.36 ± 0.86	5.89 ± 1.10	0.545

Data are presented as mean ± SD of three independent experiments performed in triplicate. Independent sample t-test was used to assess differences. Significance was reached when $p < 0.05$.

Table S10. GR_{50} values of PANCO09b and PANCO11b growing in conventional versus physiological media exposed to gemcitabine, paclitaxel, SN-38, 5-FU, and oxaliplatin.

**Cell culture**	**Drugs**	**IC_{50} (μM) ± SD**		***p*-value**
		Adv.DMEM/F-12	Plasmax	
**PANCO09b**	**Gemcitabine**	$5.33 * 10^{-3} \pm 1.04 * 10^{-3}$	$9.11 * 10^{-3} \pm 4.55 * 10^{-3}$	0.239
	Paclitaxel	$1.81 * 10^{-3} \pm 0.66 * 10^{-3}$	$2.11 * 10^{-3} \pm 0.66 * 10^{-3}$	0.605
	SN-38	$5.56 * 10^{-3} \pm 2.19 * 10^{-3}$	$6.34 * 10^{-3} \pm 2.72 * 10^{-3}$	0.392
	5-FU	17.45 ± 2.62	35.75 ± 19.02	0.31
	Oxaliplatin	11.67 ± 2.47	15.03 ± 8.22	0.535
**PANCO11b**	Gemcitabine	$7.42 * 10^{-3} \pm 1.75 * 10^{-3}$	$6.30 * 10^{-3} \pm 0.651 * 10^{-3}$	0.357
	Paclitaxel	$3.48 * 10^{-3} \pm 0.99 * 10^{-3}$	$2.26 * 10^{-3} \pm 0.74 * 10^{-3}$	0.164
	SN-38	$9.82 * 10^{-3} \pm 0.71 * 10^{-3}$	$5.18 * 10^{-3} \pm 1.83 * 10^{-3}$	0.015
	5-FU	44.85 ± 12.23	30.20 ± 14.28	0.563
	Oxaliplatin	28.65 ± 15.49	13.30 ± 3.85	0.174

Data are presented as mean ± SD of three independent experiments performed in triplicate. Independent sample t-test was used to assess differences. Significance was reached when $p < 0.05$.
